# Antibiotic profiles and draft genome sequences of four *Shigella flexneri* recovered from untreated community wastewater samples in Ghana

**DOI:** 10.1128/mra.00082-26

**Published:** 2026-07-10

**Authors:** Beverly Egyir, Alex Awentirim Akayiri, Emmanuel Ohene Kyei McKeown, Daniel Kwaku Baka, William Boateng, Christian Owusu-Nyantakyi, Grebstad Rabbi Amuasi, Quaneeta Mohktar, Gabriella Acquah, Maa Lankai Quaye, Alfred Bortey, Emmanuel Gberbi, Angella Yayra Kampo, Agnes Oclu, Justice Danso, Blessing Kofi Adu Tabi, Rhodalyn Tagoe, Evangeline Obodai, Christa Twyford Gibson, Pernille Nilsson, Rene S. Hendriksen, Bright Adu, John Kofi Odoom

**Affiliations:** 1 Department of Bacteriology, Noguchi Memorial Institute for Medical Research, University of Ghana 58835, Accra, Ghana; 2 Department of Biochemistry, Cell and Molecular Biology, University of Ghana 58835, Accra, Ghana; 3 Department of Virology, Noguchi Memorial Institute for Medical Research, University of Ghana 58835, Accra, Ghana; 4 Technical University of Denmark, National Food Institute 5205, Kgs. Lyngby, Denmark; 5 Department of Immunology, Noguchi Memorial Institute for Medical Research, University of Ghana 58835, Accra, Ghana; University of Maryland Baltimore, Baltimore, Maryland, USA

**Keywords:** *Shigella flexneri*, Ghana, wastewater, antimicrobial resistance, Africa, genome

## Abstract

Genomic data on clinically relevant bacteria of public health concern from sub-Saharan Africa are limited. In this study, we present the phenotypic profiles and genomic characteristics of four *Shigella flexneri* isolates recovered from untreated community wastewater samples in Ghana.

## ANNOUNCEMENT

Wastewater surveillance combined with whole-genome sequencing enables high-resolution monitoring of antimicrobial resistance and supports public health responses (1–7). In Africa, *Shigella flexneri* isolates show widespread extended-spectrum β-lactamase (ESBL) production and high levels of multidrug and carbapenem resistance (8). Here, we report on the draft genome sequences and antibiogram of four *S*. *flexneri* recovered from untreated community wastewater samples.

A total of 277 untreated community wastewater samples were archived from 13 sentinel sites across seven regions in Ghana. These samples were concentrated at the Virology Department of the NMIMR and stored in 2 mL cryovials for poliovirus detection. The 277 archived concentrates were then retrieved and cultured on MacConkey agar (Oxoid, UK) and incubated at 37°C for 18–24 hours. The isolates obtained were subcultured onto nutrient agar (Oxoid, UK) and identified to the species level using matrix-assisted laser desorption/ionization time-of-flight mass spectrometry (MALDI-TOF MS) (Bruker Daltonics, Germany). Antimicrobial susceptibility testing was performed on isolates using the Kirby-Bauer disk diffusion method with 13 antimicrobial agents. Detection of extended-spectrum β-lactamase production was performed using the double-disk diffusion method. Measured zone sizes were interpreted using CLSI (2024) guidelines (9).

DNA extraction and purification were performed using the QIAamp DNA Mini Kit (QIAGEN, Hilden, Germany). Quantification of DNA was done using a Qubit 4.0 fluorometer (Thermo Fisher Scientific, USA). Libraries were prepared using the Illumina DNA Preparation (M) Tagmentation Kit (Illumina Inc., San Diego, CA, USA) according to the manufacturer’s instructions. The process included the enzymatic fragmentation of DNA using bead-linked transposons, index addition, amplification, and bead-based size selection. Library fragment sizes and concentrations were assessed using the Agilent 2100 Bioanalyzer (Agilent Technologies, USA) and quantitative PCR (KAPA SYBR Fast qPCR kit), respectively. Libraries were normalized to 2 nM, pooled, and then sequenced using 2 × 150 bp paired-end chemistry on the Illumina NextSeq 2000 system (Illumina Inc., San Diego, CA, USA). Indexes and reads with quality scores <20 were trimmed using Trimmomatic v0.39 (10), quality-checked with FastQC v1.0 (11), and assembled using Unicycler v0.5.0 (12). The assemblies were evaluated using Quast v5.2.0 (13). All genomes met established quality thresholds (*Q* ≥ 20, ≥20× coverage, and contig length ≥200 bp). Species confirmation, virulence profiling, plasmid identification, and multilocus sequence typing were performed using KmerFinder v3.0.2 (14), VirulenceFinder v2.0.5 (15), PlasmidFinder v2.0.1 (16), and MLST v2.0.9 (17), respectively. Antibiotic resistance was determined using ResFinder v4.7.2 (18) and CARD v4.0.1 (19). Genome annotation was performed using NCBI PGAP v6.10 (20). Default settings were utilized except where mentioned. Table 1 presents a summary of the results.

**TABLE 1:** Antimicrobial resistance profiles and genomic characteristics of isolates^*a*^

Characteristics	Isolate information			
Sample ID	470_24_S308	543_24_S307	511_23_S251	636_23_S239
Strain ID	SF001	SF002	SF003	SF004
Geographical location	Nima Freetown (5.58° North Latitude and 0.2° West Longitude)	Ahinsan (6.66° North Latitude and −1.6° West Longitude)	Sunyani Zongo (7.34° North Latitude and −2.32° West Longitude)	Shiabu (5.53° North Latitude and −0.25° West Longitude)
Antimicrobial agents (Zone diameters/mm)				
Amikacin (30 µg)	20 (S)	20 (S)	18 (I)	18 (I)
Ampicillin (10 µg)	6 (R)	6 (R)	6 (R)	6 (R)
Cefotaxime (30 µg)	10 (R)	10 (R)	10 (R)	10 (R)
Cefoxitin (30 µg)	6 (R)	26 (S)	25 (S)	8 (R)
Ceftazidime (30 µg)	18 (I)	17 (R)	18 (I)	6 (R)
Cefuroxime (30 µg)	6 (R)	6 (R)	6 (R)	6 (R)
Chloramphenicol (30 µg)	18 (S)	24 (S)	26 (S)	25 (S)
Ciprofloxacin (5 µg)	19 (R)	20 (R)	14 (R)	21 (R)
Gentamicin (10 µg)	19 (S)	17 (I)	18 (S)	15 (I)
Meropenem (10 µg)	22 (I)	30 (S)	34 (S)	11 (R)
Norfloxacin (10 µg)	23 (S)	15 (I)	20 (S)	20 (S)
Tetracycline (30 µg)	6 (S)	7 (S)	6 (R)	7 (R)
Trimethoprim- Sulfamethoxazole (25 µg)	6 (R)	6 (R)	6 (R)	28 (S)
ESBL	Positive	Positive	Positive	Negative
Resistance genes				
Aminoglycosides	*aph (6)-ld*, *aph(3^II^)-lb*, and *aadA5*	ND	*aph (6)-Id* and *aph(3'')-Ib*	ND
β-Lactams	*bla_CTX-M-15_*	*bla_CTX-M-15_*	*bla_CTX-M-15_* and *bla_TEM-1B_*	*bla_TEM-1B_*
Folate pathway antagonists	*sul2*, *sul1*, and *dfrA17*	ND	*sul2* and *dfrA14*	ND
Macrolides	*erm(B*) and *mph(A*)	ND	ND	ND
Quinolones	*qnrD1*, *qnrS1*, *emrR*, and *emrB*	*qnrS1*, *emrR*, *emrA*, *emrB*, and *mdtB*	*qnrS1*, *emrB*, *emrR*, and *mdtH*	*qnrS1*, *emrB*, and *emrR*
Tetracyclines	*tet(A)* and *emrY*	*tet(A*)	*tet(A)* and *emrY*	*tet(A*)
Nitroimidazole	*msbA*	*msbA*	*msbA*	*msbA*
Peptide		*bacA* and *pmrF*	*pmrF*	*yojI*, *pmrF*, and *bacA*
Disinfecting agents and antiseptics	*qacEdelta1*	ND	ND	ND
Phosphonic acid	*mdtG*	ND	*mdtG*	*mdtG*
Multiple drugs^*b*^	*acrS*, *gadX*, *mdtE*, *evgA*, *acrA*, *acrB*, *cpxA*, and *marA*	*acrS*, *acrE*, *acrA*, *acrB*, *mdtN*, *mdtP*, *cpxA*, *gadX*, *mdtF*, *mdtE*, and *marA*	*evgA*, *marA*, *acrE*, *acrS*, *mdtE*, *acrB*, *acrA*, and *cpxA*	*mdtP*, *mdtN*, *acrE*, *acrA*, *acrB*, *cpxA*, *gadX*, *mdtF*, *mdtE*, *marA*, and *evgA*
Plasmids	Col3M, IncFIA, IncFIB, IncFII, and IncR	ND	IncY	IncY
Virulence genes	*anr*, *capU*, *csgA*, *fdeC*, *gad*, *hha*, *hlyE*, *iss*, *nlpl*, *sitA*, *terC*, *traT*, *yehA*, *yehB*, *yehC*, and *yehD*	*capU*, *csgA*, *fdeC*, *gad*, *hha*, *hlyE*, *iss*, *nlpI*, *sitA*, *terC*, *yehA*, *yehB*, *yehC*, *yehD*, and *yghJ*	*capU*, *csgA*, *fdeC*, *fimH*, *gad*, *hlyE*, *nlpI*, *terC*, *yehA*, *yehB*, *yehC*, *yehD*, and *yghJ*	*clpK1*, *csgA*, *fdeC*, *fimH*, *gad*, *hha*, *hlyE*, *nlpI*, *sitA*, *terC*, and *yghJ*
Genome size (bp)	4,743,972	4,586,942	4,817,493	4,510,260
Sequence type	ST1598	ST1421	ST224	ST46
N_50_ (bp)	82,775	82,809	86,580	84,358
GC content (%)	50.73	50.82	50.83	50.78
No. of reads	7,576,788	7,329,186	6,642,772	8,680,358
No. of contigs	279	198	77	145
Size of largest contig (bp)	278,327	229,652	977,979	235,020
No. of coding sequences	4,473	4,263	4,430	4,186
No. of rRNAs	9	3	9	2
No. of tRNAs	80	80	80	77
Coverage (×)	241	241	208	291
BioProject accession no.	PRJNA1338482	PRJNA1338482	PRJNA1338482	PRJNA1338482
Genome accession no.	JBRNXC000000000	JBRNXB000000000	JBSCCP000000000	JBSCCM000000000
BioSample accession no.	SAMN52447488	SAMN52447489	SAMN53088219	SAMN53088222
SRA accession no.	SRR35793167	SRR35793166	SRR35980169	SRR35980168

^*a*^ R, resistant; I, intermediate; S, susceptible; ND, not detected.

^*b*^ Genes conferring resistance to multiple drug classes. The drug classes can be found at https://doi.org/10.6084/m9.figshare.31078558.

## Data Availability

The genomes were uploaded to the National Center for Biotechnology Information (NCBI) database under BioProject accession number PRJNA1338482 and assigned unique accession numbers as provided in Table 1.
